# 

**Published:** 1916-04-08

**Authors:** 


### CONTENTS.

PAGE
**Industrial Fatigue** ... ... ... ... ... 21
**Hospital Responsibilities—Direct and Indirect** 22
**Hospital and Institutional News** ... ... ... 23
The Hospital Ship *Portugal*—London County Council Wisdom—The City Coroner's Report—A Coal Seam Under a Hospital—Hospitals and the Ban on Glycerine—The Woman's Imperial Health Association—"Ptomaine" Poisoning—The Oxfordshire Tuberculosis Scheme Held Up—The Geat Northern Central Hospital—Lancaster Infirmary's Success in Litigation—A Statue to Nurse Cavell—£3,000 in Ten Minutes—Urgent Requirements at the Essex County Hospital, Colchester—Surgical Operations on School Children—The Ambulance of the Magherafelt Guardians—The Woman's Real World—This Week's Drug Market.
**A Medical Causerie** ... ... ... ... ... 27
**Another Food Fad** ... ... ... ... ... 28
**Prolonged Jaundice** ... ... ... ... ... 29
A Difficult Problem of Diagnosis.
**The Child Delinquent** ... ... ... ... ... 30
By Cecil M. Chapman, J.P.
**Gallant R.A.M.C. Officers** ... ... ... ... 30
**Hospital Expenditure in War Time** ... ... 31
The Food Bill at St. George's.
**Some Hospital Chapels** ... ... ... ... 32
The Royal National Hospital for Consumption, Ventnor.
**The Value of Child Life** ... ... ... ... 35
Danger of Excess of Economy in Child Welfare Work.

PAGE
**The Matrons' and Sisters' Department** ... ... 33
Safely Launched and in Port.
Round the Hospitals.
The Importance of the Laundry—Dresses for Hospital Children—General Smuts' Hospital—Lady Barbers—Cruelties of Idle Tongues—Are Dogs Useful in War?
**Additional Responsibility for Hospital Managers** ... ... ... ... ... ... 36
By an Experienced Lay Superintendent.
**Centenary of the Royal Waterloo Hospital** ... 36
**Parliament and the Profession** ... ... ... 37
Conscientious Objectors as Hospital Orderlies.
Reduction in Medical Fees.
Hospital Arrangements in Mesopotamia.
Venereal Disease.
Other Matters of Interest.
**Hospital Results in 1915** ... ... ... ... 38
More Early Reports of the Voluntary Hospitals.
**Gifts and Bequests** ... ... ... ... ... 38
**Passing Events and Topics** ... ... ... ... 39
**Dresses for Hospital Children** ... ... ... 40
**Personalia**... ... ... ... ... ... ... 40
**The Book Market** ... ... ... ... ... 40
**The Institutional Worker** ... ... (Suppl.) 1
Answers to March Question (No. 2).
Question for April.
Arrangements for Military Work.
Questions Invited.
Inquiries and Answers.
The Sick and in Need.
Miscellaneous.
Employment and Training.

